# Breast cancer risk factors in Turkish women – a University Hospital based nested case control study

**DOI:** 10.1186/1477-7819-7-37

**Published:** 2009-04-08

**Authors:** Vahit Ozmen, Beyza Ozcinar, Hasan Karanlik, Neslihan Cabioglu, Mustafa Tukenmez, Rian Disci, Tolga Ozmen, Abdullah Igci, Mahmut Muslumanoglu, Mustafa Kecer, Atilla Soran

**Affiliations:** 1 Istanbul University, Istanbul Medical Faculty, Department of Surgery, Capa, Istanbul, Turkey; 2 Istanbul University, Istanbul Medical Faculty, Public Health Department, Capa, Istanbul, Turkey; 3 Magee-Womens Hospital of UPMC, Pittsburgh, USA

## Abstract

**Background:**

Breast cancer has been increased in developing countries, but there are limited data for breast cancer risk factors in these countries. To clarify the risk for breast cancer among the Turkish women, an university hospital based nested case-control study was conducted.

**Methods:**

Between January 2000 and December 2006, a survey was prospectively conducted among women admitted to clinics of Istanbul Medical Faculty for examination and/or treatment by using a questionnaire. Therefore, characteristics of patients diagnosed with breast cancer (n = 1492) were compared with control cases (n = 2167) admitted to hospital for non-neoplastic, non-hormone related diseases.

**Results:**

Breast cancer risk was found to be increased in women with age (≥ 50) [95% confidence interval (CI) 2.42–3.18], induced abortion (95% CI 1.13–1.53), age at first birth (≥ 35) (95% CI 1.62–5.77), body mass index (BMI ≥ 25) (95% CI 1.27–1.68), and a positive family history (95% CI 1.11–1.92). However, decreased breast cancer risk was associated with the duration of education (≥ 13 years) (95% CI 0.62–0.81), presence of spontaneous abortion (95% CI 0.60–0.85), smoking (95% CI 0.61–0.85), breast feeding (95% CI 0.11–0.27), nulliparity (95% CI 0.92–0.98), hormone replacement therapy (HRT) (95% CI 0.26–0.47), and oral contraceptive use (95% CI 0.50–0.69). On multivariable logistic regression analysis, age (≥ 50) years (OR 2.61, 95% CI 2.20–3.11), induced abortion (OR 1.66, 95% CI 1.38–1.99), and oral contraceptive use (OR 0.60, 95% CI 0.48–0.74) were found to be associated with breast cancer risk as statistically significant independent factors.

**Conclusion:**

These findings suggest that age and induced abortion were found to be significantly associated with increased breast cancer risk whereas oral contraceptive use was observed to be associated with decreased breast cancer risk among Turkish women in Istanbul.

## Background

Breast cancer is the most common site-specific cancer, and is the leading cause of death from cancer in women [1,2]. Breast cancer incidence and mortality may have alterations in different geographical areas. Although developed countries report higher rates of breast cancer incidence and mortality, changes in the incidence of breast cancer are most dramatic in low-middle income countries (LMC) including Turkey [3]. According to World Health Organization (WHO), countries were classified into four categories regarding their resources: basic, limited, enhanced, and maximal. Turkey is a middle income country between limited and enhanced level regarding their sources [2,4,5].

Breast cancer incidence has increased in Turkey, and the estimated number of breast cancer cases was 44,253 in 2007 [6,7]. The distribution of breast cancer incidence varies significantly among different regions of Turkey due to geographical, economic, social, and cultural factors. The breast cancer incidence in Western Turkey (50/100,000 in 2000) is more than two times that of Eastern Turkey (20/100,000) due to 'Westernized' lifestyles (early menarche, late menopause, first birth >30 years of age, less breast-feeding, etc.), an ill-defined surrogate for changes in patterns of childbearing, dietary habits, exposures to exogenous hormones, and possibly other factors similar to those of women in industrialized countries [2,7-9]. Breast cancer frequency has been found to be 20% among women <40 years old in Turkey, whereas it was found to be around 5% in Western Europe and USA. There is also geographical heterogeneity regarding breast cancer survival rates in Turkey. Five year breast cancer specific survival rates are 85% and 60% in western and eastern Turkey, respectively [9].

Numerous epidemiological studies on risk factors of breast cancer have produced evidence on international variations. Many studies in the literature have reported that breast cancer is related to the reproductive life of women; such as early menarche, late menopause, nulliparity, late age at first birth, diet, physical exercise and hormone usage [9-11]. These studies are limited among women of developing countries to identify the risk factors to conduct new prevention strategies. Hence, there is a need to develop clinical practice guidelines oriented toward countries with limited financial resources. Therefore, we aimed to assess various breast cancer risk factors to identify the characteristics among Turkish women in Istanbul.

## Methods

Between January 2000 and December 2006, we conducted a large university hospital based-nested case control study among Turkish women with or without breast cancer. A survey was prospectively conducted among women in clinics of Istanbul Medical Faculty by using a questionnaire. The Istanbul University Medical Faculty hospital is one of the busiest hospitals located in Istanbul, and over 80% of the outpatients reside in Istanbul which is the biggest city in Turkey with a population of 15 million. People who live in Istanbul have migrated from all around the Turkey. Istanbul University Medical Faculty Hospital accepts new outpatients with or without doctor's referral. Therefore, one may think that the outpatient population does reflect a general outpatient population in any hospital in Turkey. The control group consisted of 2167 women, between 18 to 70 years of age, without any known chronic illnesses (e.g. hypertension, diabetes mellitus, coronary artery disease), any neoplastic and hormone related diseases, selected from the waiting area of different clinics by convenience sampling. Cases with breast cancer were either selected from patients visiting our Breast Clinic for follow-up, or from our breast cancer database with age between 18 to 70 years (n = 1492). Sampling was not performed for both case and control group. An institutional ethical committee approval was obtained before starting with the study. All interviews were conducted at the hospital. Data were collected by a face to face interview using a questionnaire form after having the informed consents signed by the participants. The questionnaire consisted of 25 questions related to general characteristics of women (age, education, social status, body mass index (BMI), smoking (current smokers), alcohol intake), menstrual and reproductive history (use of hormone replacement therapy, age at menopause, age at first birth, breast feeding) and family history of breast cancer. The data was stored by using Microsoft Access program and the statistical analyses were performed by SPSS 15.0 program (SPSS Inc, Chicago, Illinois). The body mass index was calculated as weight (kg)/height^2 ^(m^2^). The chi-square test was used in the statistical analyses to evaluate the significant factors associated with breast cancer risk by estimating the odds ratio (OR) and 95% confidence intervals (CI). Logistic regression was used to construct a multivariable model of independent factors associated with breast cancer risk. Forward stepwise regression was used for factor selection and, only factors with a frequency >10% that exhibited univariate significance levels of less than 0.05 were examined. For each factor in the model, the likelihood of breast cancer risk was estimated by the odds ratios and 95% CI. A p value of < 0.05 was considered significant in the statistical analyses.

## Results

The distribution of patients with breast cancer (n = 1492) and control cases (n = 2167) according to sociodemographic characteristics (age, education, body mass index (BMI), smoking (independent from the total time), alcohol intake and family history of breast cancer) was shown in Table 1. The distribution of patients with breast cancer (n = 1492) and control cases (n = 2167) according to menstrual and reproductive factors were shown in Table 2.

**Table 1:** The distribution of women in the control group (n = 2167) and patients with breast cancer (n = 1492) according to the factors including age, education, body mass index (BMI), smoking and alcohol intake.

Factors	women in the control group (n = 2167)	patients with breast cancer (n = 1492)
*Age (years)*		

≥ 35 <35	1625 (75.0%) 541 (25.0%)	1410 (94.5%) 82 (5.5%)
35–39	253 (11.7%)	120 (8.2%)
40–49	636 (29.3%)	410 (27.5%)
*≥ *50	737 (34.0%)	880 (58.8%)

*Education (years)*		

<13	1110 (51.2%)	891 (59.7%)

≥ 13	1057 (48.8%)	601 (40.3%)

*Body mass index (BMI-kg/m^2^)*		

<25	971 (44.8%)	534 (35.8%)

≥ 25	1196 (55.2%)	958 (64.2%)

*Smoking*		

Never	1517 (70.0%)	1141 (76.5%)

Ever	650 (30.0%)	351 (23.5%)

*Alcohol intake*		

Never	2166 (99.99%)	1489 (99.98%)

Ever	1 (0.01%)	3 (0.02%)

*Family history of breast cancer [first-degree relative(s)]*		

No	2059 (95.0%)	1384 (92.8%)

Yes	108 (5.0%)	108 (7.2%)

**Table 2:** The distribution of women in the control group (n = 2167) and patients with breast cancer (n = 1492) according to the reproductive factors.

Factors	women in the control group (n = 2167)	patients with breast cancer (n = 1492)
*Number of children*		

Nullipara	451 (20.8%)	241(16.2%)

≥ 1	1716 (79.2%)	1251 (83.8%)

≥ 4	353 (16.3%)	324 (21.7%)
1	312 (14.4%)	238 (12.4%)
2	678 (31.3%)	506 (33.9%)
3	373 (17.2%)	183 (15.8%)

*Age at first birth (years)*		

<25	1697 (78.3%)	1158 (77.6%)

25–29	353 (16.3%)	231 (15.5%)

30–34	99 (4.6%)	64 (4.3%)

≥ 35	18 (0.8%)	39 (2.6%)

*Breast feeding*		

Never	35 (1.6%)	126 (8.5%)

Ever	2132 (98.4%)	1366 (91.5%)

*Spontaneous abortion*		

Never	1540 (71.1%)	1156 (77.5%)

Ever	627 (28.9%)	336 (22.5%)

*Induced abortion*		

Never	1237 (57.1%)	750 (50.3%)

Ever	930 (42.9%)	742 (49.7%)

*Oral contraceptive use*		

Never	1565 (72.2%)	1217 (81.6%)

Ever	602 (27.8%)	275 (18.4%)

*Hormone replacement therapy*		

Never	1933 (89.2%)	1431 (95.9%)

Ever	234 (10.8%)	61 (4.1%)

*Age at menopause (years)*		

<50	1553 (71.7%)	971 (65.1%)

≥ 50	614 (28.3%)	521 (34.9%)

Patients with age (≥ 50 years) (OR 2.78, 95% CI 2.42–3.18) or induced abortion (OR 1.31, 95% CI 1.13–1.53), or age over 35 at first birth (OR 3.06, 95% CI 1.62–5.77), or BMI (≥ 25 kg/m^2^) (OR 1.46, 95% CI 1.27–1.68), or first-degree family history of breast cancer (OR 1.46, 95% CI 1.11–1.92) were more likely to have increased breast cancer risk (Table 3). Nevertheless, factors associated with decreased breast cancer risk were as following: education over 13 years (OR 0.71, 95% CI 0.62–0.81), spontaneous abortion (OR 0.71, 95% CI 0.60–0.85), smoking (OR 0.72, 95% CI 0.61–0.85), breast feeding (OR 0.17, 95% CI 0.11–0.27), nulliparity (OR 0.95, 95% CI 0.92–0.98), hormone replacement therapy (OR 0.35, 95% CI 0.26–0.47), and oral contraceptive use (OR 0.59, 95% CI 0.50–0.69) (Table 3). However, total time of breast feeding has found to no significant effect in breast cancer risk. Alcohol intake was very limited, and less than 1% of women in both groups received alcohol, and therefore, this factor was not evaluated in statistical analyses.

**Table 3:** Risk factors associated with increased or decreased risk of breast cancer.

*Factors associated with increased breast cancer risk:*	women in the control group (n = 2167) (%)	patients with breast cancer (n = 1492) (%)	OR (95%CI)	P value
Age (≥ 50) years	34.0	58.8	2.78 (2.42–3.18)	< 0.001

Induced abortion	42.9	49.7	1.31 (1.13–1.53)	< 0.001

Body mass index ≥ 25	55.2	64.2	1.46 (1.27–1.68)	< 0.001

Family history of breast cancer (first degree relative)	5.0	7.2	1.46 (1.11–1.92)	0.008

Age at first birth (≥ 35 years)	0.9	2.6	3.06 (1.62–5.77)	< 0.001

*Factors associated with decreased breast cancer risk:*				

Education (≥ 13 years)	48.8	40.3	0.71 (0.62–0.81)	< 0.001

Spontaneous abortion	28.9	22.5	0.71 (0.60–0.85)	< 0.001

Smoking	29.9	23.5	0.72 (0.61–0.85)	< 0.001

Breast feeding	98.4	91.5	0.17 (0.11–0.27)	< 0.001

Oral contraceptive use	27.8	18.4	0.59 (0.50–0.69)	< 0.001

Nullipara	20.8	16.2	0.95 (0.92–0.98)	< 0.001

Hormone replacement therapy	10.8	4.1	0.35 (0.26–0.47)	< 0.001

The significant risk factors with a distribution frequency >10% including age ≥ 50, induced abortion, BMI ≥ 25, education ≥ 13 years, spontaneous abortion, smoking, breast feeding, oral contraceptive use and nulliparae were further evaluated in the multivariable logistic regression analyses. Among these factors, age (≥ 50) years (OR 2.61, 95% CI 2.20–3.11), induced abortion (OR 1.66, 95% CI 1.38–1.99), and oral contraceptive use (OR 0.60, 95% CI 0.48–0.74) were found to be associated with breast cancer risk as statistically significant independent factors (Table 4).

**Table 4:** Results of logistic regression model for factors associated with breast cancer risk.

Factors*	OR (95%CI)	P value
Age (≥ 50) years	2.61 (2.20–3.11)	< 0.001

Induced abortion	1.66 (1.39–1.98)	< 0.001

Oral contraceptive use	0.60 (0.48–0.74)	< 0.001

*The significant risk factors in Table 3 with a distribution frequency >10% including age ≥ 50, induced abortion, BMI ≥ 25, education ≥ 13 years, spontaneous abortion, smoking, breast feeding, oral contraceptive use and nullipara were further evaluated in the multivariable logistic regression analyses.

## Discussion

We are aware that this hospital based study has some potential biases such as selection biases (non response bias, hospital admission bias, exclusion bias), and information bias (interview bias, recall bias, reporting bias). One form of hospital admission bias, the problem is that hospitalized individuals are more likely to suffer from many illnesses or symptoms. Thus they are probably not representative of the target population. On the other hand, Istanbul University Istanbul Medical Faculty Hospital is one of the busiest hospitals located in Istanbul and over 80% of the outpatients reside in the Istanbul area, which has a population of 15 million. This hospital accepts new outpatients with or without doctor's referral. Therefore, one may think that the outpatient population may potentially reflect a general outpatient population in this hospital in Turkey, and the control group consisted of women with non-neoplastic and non-hormone related illnesses selected from the waiting area of different clinics. Although the study was not population-based, patients diagnosed and treated in a large Istanbul Medical Faculty Hospital were included that limited any potential biases related with the treatment. Furthermore, strength of this study is its relatively large size, which provided reasonably stable risk estimates.

The incidence of breast cancer increases with age, doubling about every 10 years until the menopause. McPherson et al reported that, of every 1000 women aged 50, two will recently have had breast cancer diagnosed and about 15 will have had a diagnosis made before the age of 50, giving a prevalence of breast cancer of nearly 2% [12]. Vogel et al suggested that, the risk of breast cancer increases among women older than 50 years of age especially who have benign breast disease, especially those with atypical ductal or lobular hyperplasia [13]. This study also showed that an age ≥ 50 year has effect on increased breast cancer risk significantly both in univariate and multivariable analyses.

Our study revealed that spontaneous abortion was associated with the decreased risk of breast cancer in univariate analysis whereas induced abortion was associated with increased breast cancer risk in both univariate and multivariable analyses. Some previous studies suggested that, induced or spontaneous abortions were associated with either increased or decreased risk of breast cancer, or no associations could be found with breast cancer risk for these factors [14-17]. Paoletti et al reported that a history of spontaneous abortion was not associated with breast cancer risk, although the risk was slightly increased with repeated miscarriages [18]. That study also showed that spontaneous abortion was associated with decreased risk of premenopausal breast cancer followed by an increased risk of postmenopausal breast cancer [18]. In the EPIC study, the relative risk of breast cancer of women who did not report any previous spontaneous abortions, was significantly found to be increased compared to those who reported two or more spontaneous abortions than for those reported one [19]. In the Iowa cohort, the age adjusted risk among women who had experienced an induced abortion was 1.1 compared to those who never had an induced abortion [20]. Furthermore, Michels et al found a positive association between induced abortion and breast cancer risk in women younger than 50, and a negative association in older women [21]. Therefore, similar to our findings the majority of the studies reported that induced abortion was associated with increased breast cancer risk.

It was found that hormone replacement therapy (HRT) and oral contraceptive use were directly related to breast cancer risk in many epidemiologic studies [22-25]. Conversely, other studies reported that oral contraceptive use did not increase breast cancer risk [26,27]. In the present study, we found that use of oral contraceptive use was associated with decreased breast cancer risk in both univariate and multivariable analyses whereas HRT was interestingly found to be associated with decreased breast cancer risk only in univariate analysis. However, these results were not dose and duration dependent. Therefore, further studies are required to test the consistency of our findings.

Tavani et al suggested [28] that older age at first birth (≥ 30 years) was associated with increased breast cancer, our results did support their data that being equal or more than 35 years of age at first birth is associated with increased breast cancer risk in univariate analysis. Late age at first birth delays terminal duct proliferation of mammary gland, and these women may have a higher proportion of epithelial cells that are susceptible to carcinogenic insult [29].

The most well established and documented data about endocrinological factors that decrease breast cancer risk are ever having breast fed and longer durations of breast feeding [3,29-31]. Some studies showing a longer duration of breast feeding decreases breast cancer risk [30,32]. Kim et al, suggested that average duration of breast feeding for 11–12 months reduced the breast cancer risk by 54% in Korean women as opposed to the duration of 1 and 4 months [3]. Kuru et al [11] similarly showed that there was a significant association in Turkish women with breast feeding and decreased risk of breast cancer. Our data in univariate analysis also suggested that the association between decreased risk of breast cancer and breastfeeding. However, we could not find any relationship between the duration of breast feeding and risk of breast cancer.

Many studies suggest that the educational level is associated with increased risk of breast cancer [33-36]. Tavani et al [28] revealed that patients with breast cancer were significantly more educated (>13 years) than controls [28]. This increased risk in these women may be due to the western life style in these women associated with HRT use or dietary changes or decreased exercise, or obesity or late age at first birth or decreased breast-feeding. Contrary to these findings, our study found that education (>13 years) was associated with decreased breast cancer risk in univariate analysis. These results may be due to some cultural differences based on the fact that educated Turkish women may be less affected by western life style compared to other women in the world or due the increased awareness for cancer screening etc.

The results of epidemiological studies of the association between cigarette smoking and breast cancer risk have been inconsistent [37-39]. Several recent analyses have suggested an increased risk of breast cancer among women who smoked cigarettes for a long period of time and/or who started smoking before their first pregnancy [38,40-42]. Canadian National Breast Screening Study [43] reported that there was a statistically significant association between the duration of cigarette smoking (>40 years versus null, OR = 1.50), or the intensity of smoking (>40 cigarettes per day versus null, OR = 1.20), or the cumulative exposure (>40 pack-years versus null, OR = 1.17). Cigarette smoking appears to have antiestrogenic effects. Estrogen is well established risk factor of breast cancer. Since smokers have an earlier age at menopause [43], cigarette smoking might protect against breast cancer due to its antiestrogenic effects. In univariate analysis, our data also showed an inverse association between cigarette smoking and breast cancer risk. However, duration or intensity of smoking was not investigated in the current report that might be one of the weaknesses of this study. On the other hand, this finding should not be interpreted that women should be encouraged to smoke to decrease their breast cancer risk. It is well known that cigarette smoking has so many potential side effects associated with increased cancer risks for many other types of cancer such as lung cancer, or esophageal cancer, or laryngeal cancer etc.

One of the strongest risk factors for developing breast cancer is a family history of disease. In concordance with previous studies [44,45], we also found an increased breast cancer risk associated with first-degree family history of breast cancer (mother or sister) in univariate analysis. Similarly, BMI equal or more than 25 was associated with increased breast cancer risk in both previous reports [46-51] and our current study in univariate analysis The association of increased breast cancer risk has been especially well established for young premenopausal women related to low physical activity, and anovulation in overweight and obesity [46-51].

## Conclusion

Our findings suggest that age and induced abortion were found to be significantly associated with increased breast cancer risk whereas oral contraceptive use was observed to be associated with decreased breast cancer risk among Turkish women in Istanbul in multivariable analysis. The discrepancies between our findings and other studies in the literature might be due to the different characteristics of Turkish women that merit further investigation.

## Competing interests

The authors declare that they have no competing interests.

## Authors' contributions

VO carried out study conception and design, drafting of the manuscript. BO helped to draft the manuscript and acquisition of data, analysis and interpretation of data, she has been involved in drafting the manuscript or revising critically for important intellectual content. HK helped to acquisition of data, helped in design of the study. NC participated drafting of the manuscript, editing of final version. RD performed the statistical analysis. TO helped to acquisition of data. AI, MM and MK helped to draft manuscript. AS helped to draft and critical revision of the manuscript. All authors read and approved the manuscript.
